# Supplementary material for: Bhlhe40 Regulates Proliferation and Angiogenesis in Mouse Embryoid Bodies under Hypoxia
Source: Int J Mol Sci. 2024 Jul 12;25(14):7669. doi: 10.3390/ijms25147669 (PMC11277088; doi:10.3390/ijms25147669)
Supplement: Supplementary file 1 [file ijms-25-07669-s001.zip › ijms-3067106-supplementary.pdf]

Bhlhe40 Regulates Proliferation and Angiogenesis in Mouse Embryoid Bodies under Hypoxia

Barbara Acosta-Iborra, Ana Isabel Gil-Acero, Marta Sanz-Gómez, Yosra Berrouayel, Laura Puente-Santamaría, Maria Alieva, Luis del Peso and Benilde Jiménez

A

BrCs	Exon	sgRNA Bhlhe40	Oligo 5'-3'
97.8	3	sg5: GGGCAATGCACTCGTTAATC	CACCGGGCAATGCACTCGTTAATC<br>AAACGATTAACGAGTGCATTGCCC
86.6	2	sg4: CCCGCCTGGACTTGTACACT	CACCGCCGCCTGGACTTGTACACT<br>AAACAGTGTACAAGTCCAGGCGGC
83.5	2	sg3: TGTACCAAGTGTACAAGTCC	CACCGGTACCAAGTGTACAAGTCC<br>AAACGGACTTGTACACTTGGTACC
81.8	1	sg1: TGGAGCACGGAGACCTGTCA	CACCGGGAGCACGGAGACCTGTCA<br>AAACTGACAGGTCTCCGTGCTCCC
80.0	1	sg2: CTGGAGCACGGAGACCTGTC	CACCGTGGAGCACGGAGACCTGTC<br>AAACGACAGGTCTCCGTGCTCCAC

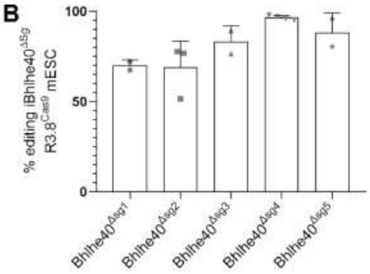

C

BrCs	Exon	sgRNA Arnt	Oligo 5'-3'
98.3	1	sg2: GTTAGCTGTAGTCGCCGCCA	CACCGTTAGCTGTAGTCGCCGCCA<br>AAACTGGCGGCGACTACAGCTAAC
97.9	2	sg4: GGGCTATTAAGCGACGGTCA	CACCGGGCTATTAAGCGACGGTCA<br>AAACTGACCGTCGCTTAATAGCCC
97.4	5'UTR	sg1: GGACGGTGCCATCTCGACCA	CACCGGACGGTGCCATCTCGACCA<br>AAACTGGTCGAGATGGCACCGTCC
95.9	2	sg3: GACATCAGATGTACCATCGC	CACCGACATCAGATGTACCATCGC<br>AAACGCGATGGTACATCTGATGTC

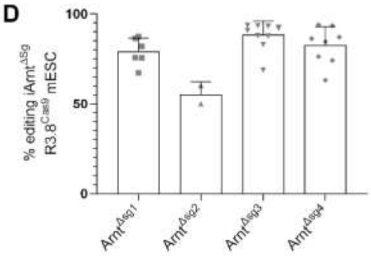

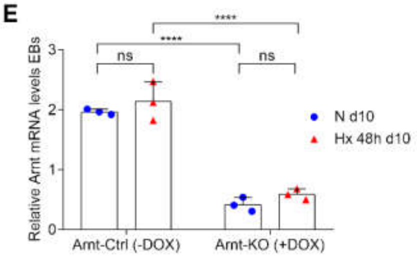

Supplementary Figure S1. Sequence of single guide RNAs for the generation of Bhlhe40 and Arnt knockout mESC lines and editing efficiency: (**A**) Sequence of the sgRNAs used for the generation of inducible CRISPR Bhlhe40-knockout cell lines (iBhlhe40$^{\Delta Sg}$ R3.8^{Cas9}). (**B**) The percentage of editing using different sgRNAs specific to Bhlhe40 was determined by qRT-PCR. Each symbol corresponds to one experiment, the bars represent the mean value of 2-4 experiments, and SD is shown. (**C**) Sequence of the sgRNAs used for the generation of inducible CRISPR Arnt-knockout cell lines (iArnt$^{\Delta Sg}$ R3.8^{Cas9}). (**D**) The percentage of editing using different sgRNAs specific to Arnt was determined by qRT-PCR. Each symbol corresponds to one experiment, the bars represent the mean value of 2-8 experiments, and SD is shown. (**E**) The efficiency of Arnt knockout in EBs after 10 days of differentiation was analyzed by qRT-PCR in normoxic (21% O_2) and 48 hours hypoxic (1% O_2) conditions. Each symbol corresponds to one experiment, and the bars represent the mean of 3 experiments. Statistical significance was determined by two-way ANOVA using Tukey's multiple comparisons post-test (ns = not significant; ****$p < 0.0001$), and SD is shown.

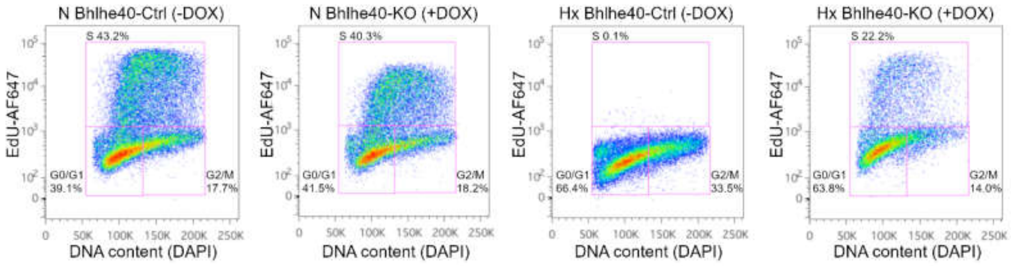

Supplementary Figure S2. Bhlhe40 knockout prevents hypoxia-induced cell cycle arrest in EBs: Additional experiment for flow cytometry analysis of EdU incorporation in total cells disaggregated from EBs of the different experimental conditions. Plots of EdU-Alexa Fluor 647 versus DNA content DAPI are shown for Bhlh40-Ctrl (–DOX), Bhlhe40-KO (+DOX), Arnt-Ctrl (–DOX) and Arnt-KO (+DOX) in normoxia (21% O_2, N) or hypoxia (1% O_2, Hx)**.** The percentage of cells in G0/G1, S, and G2/M is shown in the corresponding gating regions (magenta lines) in all experimental conditions.

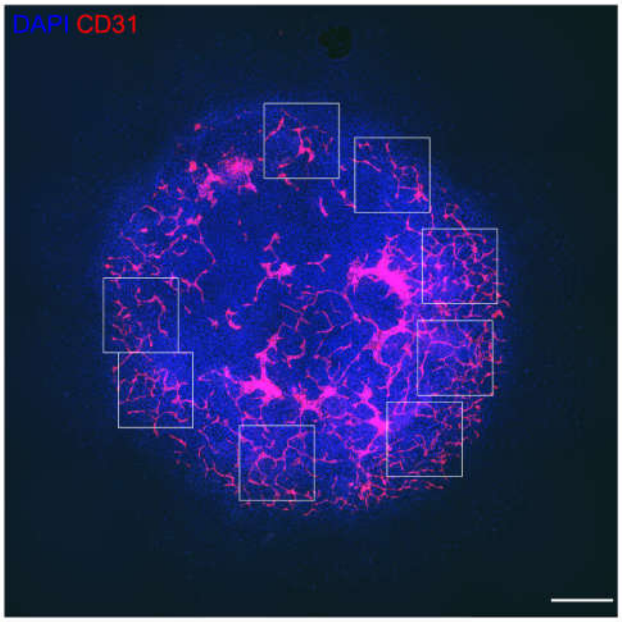

Supplementary Figure S3. A representative mosaic image of an EB showing the microscopy fields acquired to quantify the percentage of EdU+ S-phase cells in HOXD9+ progenitors and ECs in vascular structures. White squares indicate microscopy fields used for EdU quantification. Nuclei are stained with DAPI (blue), and the vascular network is stained with CD31 (red). Bar: 500 μm.

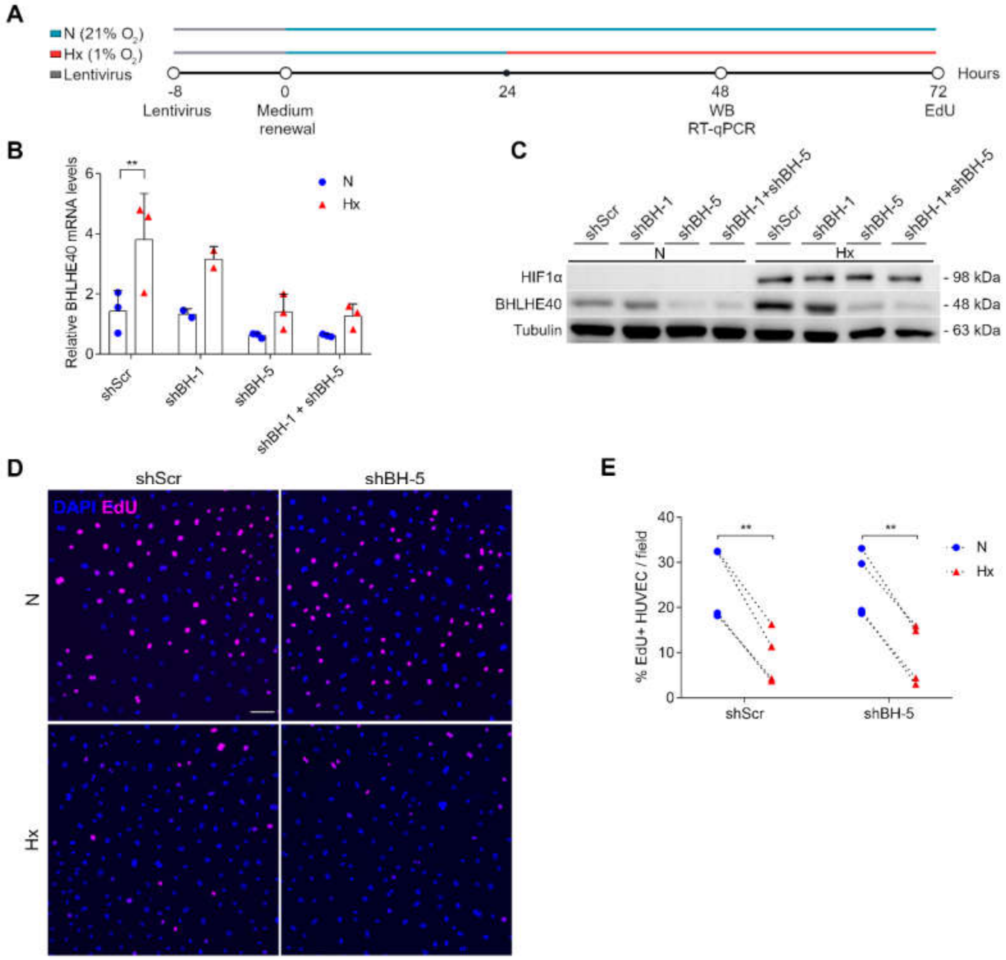

Supplementary Figure S4. BHLHE40 interference does not affect EdU incorporation in normoxic or hypoxic conditions in HUVECs: (**A**) Scheme of the experimental design. Exponentially growing HUVECs were transduced with lentivirus to express shRNAs to silence BHLHE40 expression (shBH). As a control, HUVECs were transduced with a scramble sequence that did not interfere with any human gene (shScr). Cells were exposed to normoxia (21% O_2, N, blue circles) or 48-hours hypoxia (1% O_2, Hx, red triangles). mRNA and protein levels of BHLHE40 were measured by qRT-PCR and western blot, respectively, at 24 hours. Cells were pulse-labeled with EdU during the last 3 hours to quantify the percentage of S-phase cells by microscopy. (**B**) Efficiency of BHLHE40 interference was determined by qRT-PCR. The graph represents the relative mRNA of BHLHE40 (fold over normoxia shScr) of three independent experiments. The bar represents the mean value, and SD is shown. (**C**) Efficiency of BHLHE40 interference was determined by western blot. Tubulin was used as a loading control. Stabilization of HIF1α in hypoxic conditions is also shown. (**D**) A representative microscopy image of each experimental condition (shScr and shBH-5 (the most effective shRNA)) in normoxia (21% O_2, N) or 48 hours hypoxia (1% O_2, Hx) is shown. Nuclei were visualized by DAPI staining (shown in blue). EdU^+ nuclei were visualized by Click-iT (shown in magenta). Bar: 100 µm. (**E**) Percentage of EdU^+ cells/field in normoxia (21% O_2, N, blue circles) or 48 hours hypoxia (1% O_2, Hx, red triangles) in four independent experiments. 16 fields were quantified per experimental condition, and each symbol corresponds to the mean value of one experiment ($1.2 \times 10^4 – 2.0 \times 10^4$ cells were quantified per experimental condition). Statistical significance was determined by two-way ANOVA using Sidak's multiple comparisons post-test (**$p < 0.01$).

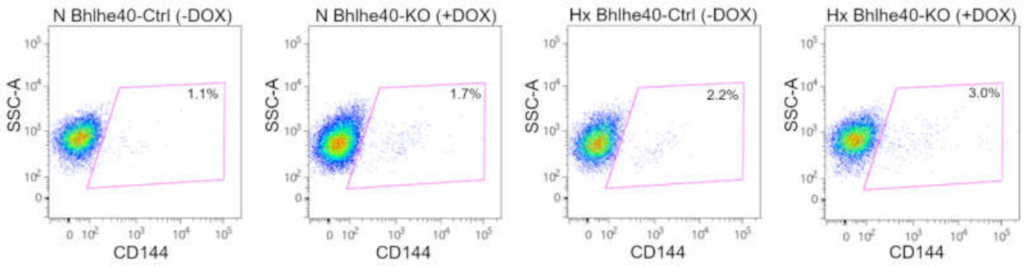

Supplementary Figure S5. Bhlhe40 knockout potentiates angiogenesis in EBs: Additional experiment for flow cytometry analysis of the effect of Bhlhe40 knockout in EBs in normoxia (21% O_2, N) and hypoxia (1% O_2, Hx). Plots of the percentage of $CD144^+$ cells in EBs for Bhlh40-Ctrl (–DOX), Bhlhe40-KO (+DOX) in normoxia (21% O_2, N) or hypoxia (1% O_2, Hx) are shown. The region of CD144-labeled cells is indicated by the magenta line and the percentage of $CD144^+$ cells is shown inside the corresponding gating regions.

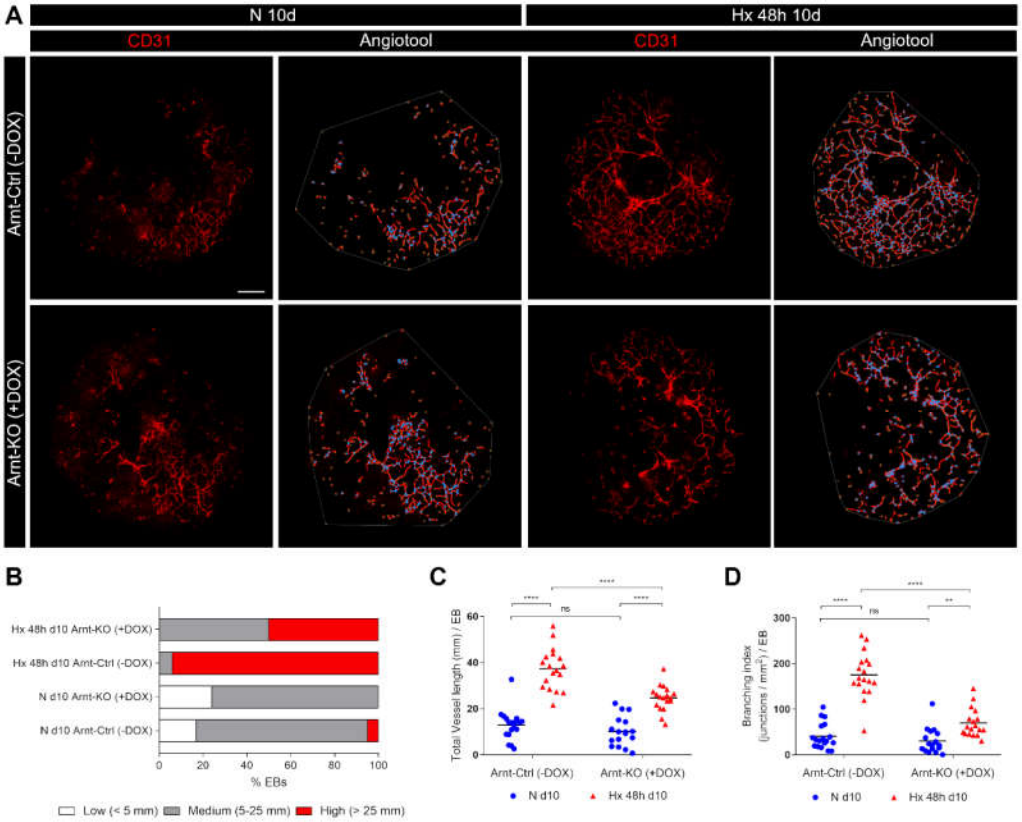

Supplementary Figure S6. Arnt knockout prevented hypoxia-induced angiogenesis in EBs: (**A**) A representative image of the EB vascular network visualized by CD31 staining (red, left images) for each experimental condition (N 10d Arnt-Ctrl (–DOX), Hx 48 hours 10d Bhlhe40-Ctrl (–DOX), N 10d Bhlhe40-KO (+DOX), and Hx 48 hours 10d Bhlhe40-KO (+DOX)) is shown. Bar: 500 μm. The AngioTool skeleton of the EB vascular network is shown by red lines, and the branching points are indicated by blue dots (right images). The area occupied by the vessel network in the EBs (AngioTool explant area parameter) is indicated by a grey line. Bar: 500 μm. (**B**) Angiogenesis was quantified using AngioTool analysis software (https://ccrod.cancer.gov/confluence/display/ROB2/Home), and the percentage of EBs with low (< 5 mm length, white), medium (5 - 25 mm length, gray), and high (> 25 mm length, red) angiogenesis is represented for each experimental condition. 11 - 18 EBs were quantified for each experimental condition. A Chi-square analysis showed that the distribution of vessel length was significantly different among conditions (χ^2_6= 263.6, ****p < .0001). (C-D) The main parameters obtained with AngioTool were represented: (**C**) Total vessel length (mm) / EB and (**D**) Branching index (junctions / mm^2) / EB. Each symbol corresponds to one EB, and the horizontal line represents the mean of all EBs quantified per experimental condition. Statistical significance was determined by unpaired t-test (ns = not significant; **p <0.01; ****p < 0.0001) (C) and by Mann-Whitney test (D).
